# Combined Effects of Confinement and Macromolecular Crowding on Protein Stability

**DOI:** 10.3390/ijms21228516

**Published:** 2020-11-12

**Authors:** Murial L. Ross, Jeffrey Kunkel, Steven Long, Prashanth Asuri

**Affiliations:** Department of Bioengineering, Santa Clara University, Santa Clara, CA 95053, USA; mross@scu.edu (M.L.R.); jkunel@scu.edu (J.K.); slong1@scu.edu (S.L.)

**Keywords:** macromolecular crowding, confinement, protein activity and stability, in vitro platforms

## Abstract

Confinement and crowding have been shown to affect protein fates, including folding, functional stability, and their interactions with self and other proteins. Using both theoretical and experimental studies, researchers have established the independent effects of confinement or crowding, but only a few studies have explored their effects in combination; therefore, their combined impact on protein fates is still relatively unknown. Here, we investigated the combined effects of confinement and crowding on protein stability using the pores of agarose hydrogels as a confining agent and the biopolymer, dextran, as a crowding agent. The addition of dextran further stabilized the enzymes encapsulated in agarose; moreover, the observed increases in enhancements (due to the addition of dextran) exceeded the sum of the individual enhancements due to confinement and crowding. These results suggest that even though confinement and crowding may behave differently in how they influence protein fates, these conditions may be combined to provide synergistic benefits for protein stabilization. In summary, our study demonstrated the successful use of polymer-based platforms to advance our understanding of how in vivo like environments impact protein function and structure.

## 1. Introduction

Until recently, most in vitro investigations of protein function and stability have been based on simple buffer systems that do not closely mimic the complex in vivo cellular environments [1,2,3,4]. Inside cells, proteins exist and function in highly crowded and compartmentalized environments that have a significant impact on several of their functions, including diffusion, enzymatic activity, protein–protein interactions, and folding, unfolding, and refolding [5,6]. Several studies have proposed the use of high concentrations of natural and synthetic macromolecules to study crowding [7,8,9,10], and encapsulating proteins within the pores of silica, polyacrylamide, or other hydrogels to study confinement [11,12,13,14,15,16,17]. These studies have contributed to the mechanistic understanding of how proteins behave in vivo, as well as to unravel the differences between how confinement and crowding influence protein function [14,16,17]. The latter outcome is significant because confinement and crowding are often used synonymously, even though these environments are distinct in how they exert their influence on protein fates. Crowding refers to effects of volume exclusion by soluble macromolecules, while confinement refers to the effects due to the presence of a fixed, impenetrable boundary [18,19]. Several studies, both theoretical and experimental, have demonstrated that this distinction is important in how the two environments may affect protein structure and function [14,18,19].

While the addition of macromolecules to protein solutions has enabled researchers to more closely simulate in vivo cellular environments, the cytosol is a complex mixture of macromolecules and cannot be fully captured by the use of a single crowding agent. To better reproduce the cellular complexity, recent studies have demonstrated the successful use of a mixture of crowding agents. For example, Zhou et al. developed conditions for mixed macromolecular crowding using both protein- and polysaccharide-based crowding agents to better simulate the intracellular environments, and demonstrated that mixed macromolecular crowding can play a significant role in refolding rates, stability, and amyloid formation of lysozyme, compared with single crowding agents [20,21]. Using mixtures of crowders of different shapes and sizes, Shahid et al. showed that mixed macromolecular crowding can exert a greater effect than the sum effects of the individual crowders, offering further support to the hypothesis that the composition of crowders, not just their final concentrations, significantly influence protein function and stability [22,23]. Our own previous work has revealed that these observations can be extended to protein confinement; we demonstrated that two different polymer networks may be combined to vary the degree of protein confinement and influence the functional and structural stability of proteins [15].

In this study, we describe our attempts to build on previous research and develop conditions to study the combined effects of confinement and crowding on protein stability [17,24,25,26]. These studies are relevant as recent studies have shown that investigations of protein fates in the combined presence of confinement and crowding can shed light on the underlying mechanisms of both single- and multi-domain protein folding and stability in vivo [17,26]. We investigated the combined effects of confinement and crowding on the functional and structural stability of two model proteins, horseradish peroxidase (HRP) and β-galactosidase (β-gal), using agarose scaffolds incorporating various amounts of dextran, an inert polysaccharide. The porosity of agarose, and thereby the degree of protein confinement, can be modified by simply changing the concentration of agarose, as demonstrated previously [15,27,28]. Several research studies have demonstrated that dextran, either alone or in combination with other crowding agents, has pronounced positive effects on protein structure and function [9,23,29,30]. Consistent with previous studies, our data demonstrated a strong influence of crowding on the thermal stability of proteins encapsulated within the pores of the agarose gels. The stabilization effects of crowding, however, were dependent on the degree of protein confinement, indicating the importance of studying different combinations of confinement and crowding on protein fates.

## 2. Results

### 2.1. Protein Confinement Increases Thermal Stability of Proteins

**Figure 1:**
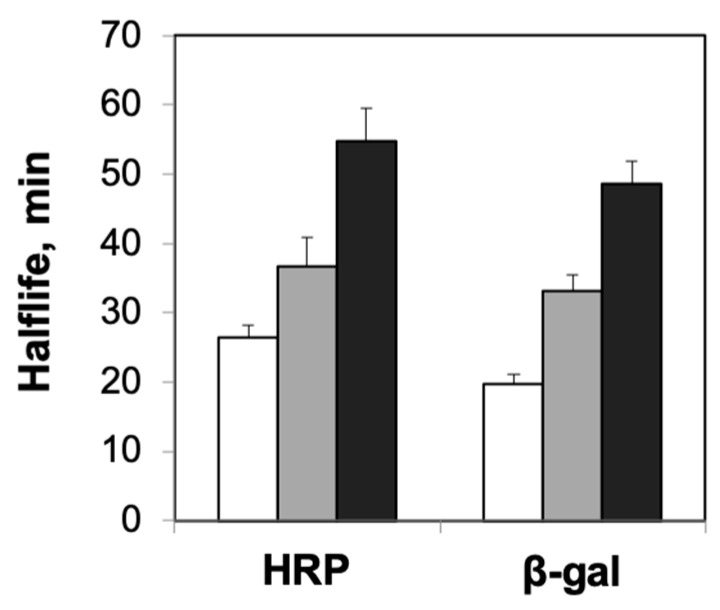
Thermal stability of horseradish peroxidase (HRP) and β-galactosidase (β-gal) encapsulated in agarose gels. Estimated half-lives of solution-phase enzymes (white bars) and enzymes encapsulated in 0.5% agarose (gray bars) and 2% agarose (black bars) at elevated temperatures. Error bars indicate the standard deviation of triplicate measurements.

**Figure 2:**
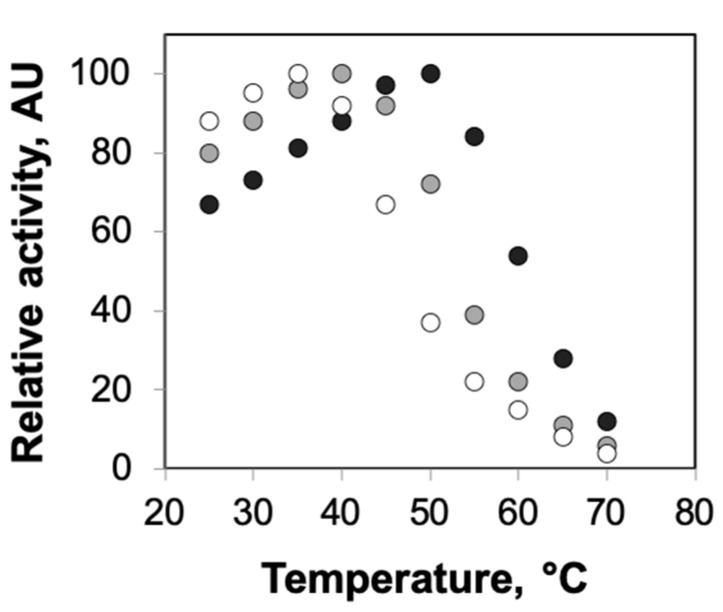
Optimum temperatures of HRP encapsulated in agarose gels. The enzyme activities of solution-phase HRP (white circles) and HRP encapsulated in 0.5% agarose (gray circles) and 2% agarose (black circles) were measured at different temperatures ranging from 25 to 70 °C. Each data point represents an average of triplicate measurements, with standard deviation <15%.

First, we performed experiments to explore the effects of protein confinement on the thermal stability of the model enzymes, horseradish peroxidase and β-galactosidase. For this, we compared the half-lives of the enzymes encapsulated in different concentrations of agarose with solution-phase enzymes at temperatures that lead to rapid loss in their function [31,32,33,34]. We used 0.5% and 2% agarose gels for these studies as previous studies have reported significant differences in the average pore radii for these concentrations (ca. 300 nm for 0.5% and ca. 75 nm for 2% agarose gels) [27,28]. The half-lives of agarose-encapsulated HRP were ca. 37 min for 0.5% agarose and ca. 55 min for 2% agarose, showing the clear dependence of agarose concentration on enzyme stability (Figure 1). Furthermore, these results indicated a statistically significant enhancement in thermal stability for agarose-encapsulated HRP relative to solution-phase HRP, whose half-life was measured to be ca. 26 min. These effects of agarose encapsulation on enzyme stability, i.e., greater enhancements in stability at higher concentrations of agarose, were not unique to HRP, but also observed for β-gal (Figure 1). The observed influence of agarose concentration on the stability of the encapsulated enzymes was expected, as our previous investigations reported higher tolerance of enzymes encapsulated in 2% agarose to chemical denaturants compared with those encapsulated in 0.5% agarose [15]. When we explored the effects of confinement on the optimum temperatures of the enzymes, we observed that the enhanced thermal stability of agarose-encapsulated enzymes was also reflected by the higher optimal temperatures for enzymes encapsulated in agarose (Figure 2).

### 2.2. Additive Effects of Confinement and Crowding on Protein Stability

**Figure 3:**
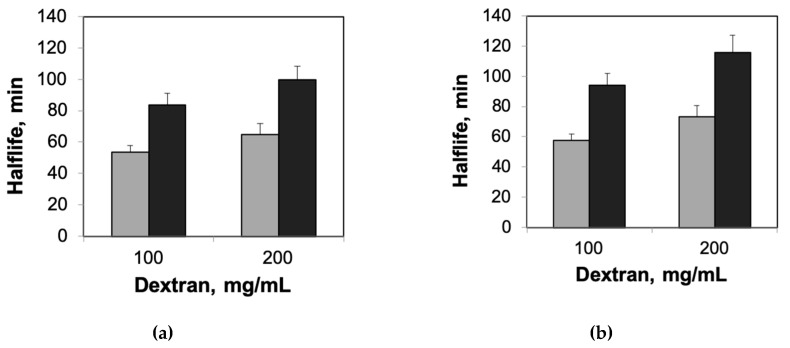
Thermal stability of enzymes encapsulated in agarose–dextran hybrid gels. Estimated half-lives of enzymes encapsulated in 0.5% agarose (gray bars) and 2% agarose (black bars) gels incorporating 100 mg/mL and 200 mg/mL dextran at elevated temperatures. (**a**) HRP and (**b**) β-gal. Error bars indicate the standard deviation of triplicate measurements.

Next, we designed experiments to explore the combined roles of confinement and crowding on the thermal stability of enzymes. We used dextran for these experiments, as dextran has been shown to increase protein stability, both on its own and in combination with other crowding agents [9,23,29,30]. Our experiments confirmed a concentration-dependent increase in protein stability for HRP and β-gal in the presence of dextran (Figure S1). When we repeated the experiments using agarose–dextran hybrid gels (Figure 3), we observed higher increases in half-lives of enzymes encapsulated in agarose gels containing dextran (compared with those encapsulated in agarose gels without dextran, Figure 1). Moreover, we observed that the relative increases in the half-lives in the presence of dextran were higher for agarose-encapsulated enzymes relative to solution-phase enzymes in the presence of dextran (Table 1). The observed non-additive effects of confinement and crowding on the enzymes encapsulated in agarose–dextran hybrid gels were consistent with previous investigations that demonstrated greater stabilizing effects of mixtures of crowding agents when compared with individual crowding agents [22,23]. It is important to note that the differences in relative increases in the half-lives due to the presence of dextran did not scale proportionally with agarose gel concentration (Table 1). However, it is reasonable to expect a saturation in the combined benefits of confinement and crowding, given the already high degree of confinement and enzyme stabilization in 2% agarose gels.

### 2.3. Crowding Enhances the Optimum Temperature of Confined Proteins

**Figure 4:**
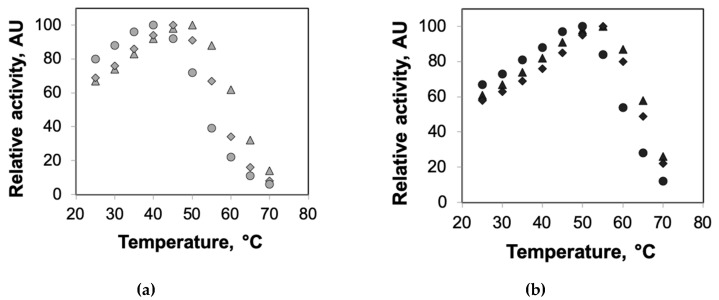
Optimum temperatures of HRP encapsulated in agarose–dextran hybrid gels. The enzyme activities of HRP encapsulated in agarose (circles) and agarose incorporating 100 mg/mL (diamonds) and 200 mg/mL (triangles) dextran—(**a**) 0.5% agarose (gray markers) and (**b**) 2% agarose (black markers)—were measured at different temperatures ranging from 25 °C to 70 °C. Each data point represents an average of triplicate measurements, with standard deviation <15%.

We also explored the effects of crowding on the optimum temperatures of the agarose-encapsulated enzymes. For this, the enzyme activities of HRP encapsulated in agarose incorporating different concentrations of dextran were measured at different temperatures and compared to those encapsulated in agarose gels without dextran. The results of these experiments were in agreement with our prior thermal deactivation experiments. We observed increases in the optimum temperatures (ca. 5 °C shift in optimum temperature) of agarose-encapsulated HRP upon the addition of dextran (Figure 4). These experiments also demonstrated a saturation in the combined benefits of confinement and crowding for enzymes encapsulated in 2% agarose.

### 2.4. Crowder Concentration and Not Size Plays a more Significant Role on Protein Stability

To further explore the combined roles for confinement and crowding on protein stability, we performed the thermal stability experiments for enzymes encapsulated in agarose–dextran gels prepared using different molecular weights of dextran (~500 kDa in Table 1 and ~150 kDa in Table 2). Specifically, we were interested in understanding the relative roles of dextran molecular weight and concentration on the enhancements in thermal stability of the confined enzymes. Consistent with aforementioned results, enzymes encapsulated in agarose–dextran hybrid gels were more thermostable than the enzymes encapsulated in agarose gels without dextran (Table 2). Furthermore, irrespective of the dextran molecular weight, the combined effects of confinement and crowding were non-additive and exhibited saturation behavior at higher concentrations of agarose (i.e., 2% agarose). These results indicated that dextran concentration and not molecular weight is a more important parameter for stability of proteins confined in agarose (Table 1 and Table 2), consistent with previous investigations that indicated volume occupied by the crowding agent (and not its size) plays an important role in protein stability [35,36,37].

### 2.5. Combined Effects of Confinement and Crowding on the Structural Stability of Proteins

**Figure 5:**
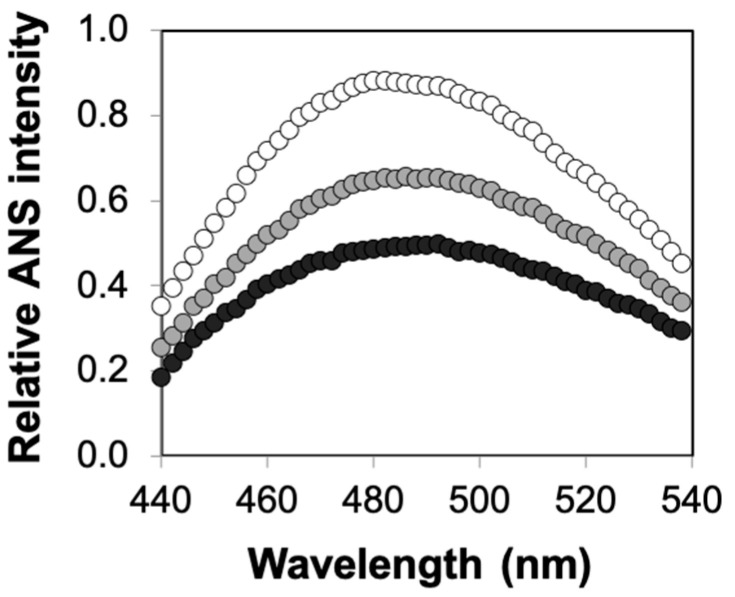
Structural stability of HRP encapsulated in agarose gels. Relative 8-anilino-1-naphthalenesulfonic acid (ANS) fluorescence intensities for solution-phase enzyme (white) and enzyme encapsulated in 0.5% agarose (gray) and 2% agarose (black) at 60 °C. Relative fluorescence intensities for the agarose–HRP formulations are reported after subtraction of the spectra of the corresponding agarose gels containing no enzyme. Each data point represents an average of triplicate measurements, with standard error <15%.

**Figure 6:**
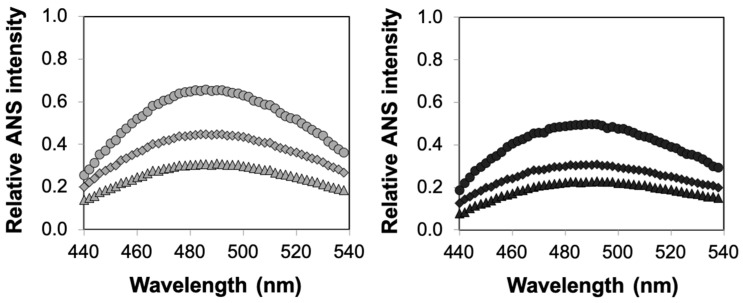
Structural stability of HRP encapsulated in agarose–dextran hybrid gels. Relative ANS fluorescence intensities for HRP encapsulated in agarose (circles) and agarose incorporating 100 mg/mL (diamonds) and 200 mg/mL (triangles) dextran at 60 °C—0.5% agarose (left, gray markers) and 2% agarose (right, black markers). Relative fluorescence intensities for the agarose–dextran HRP formulations are reported after subtraction of the spectra of the corresponding agarose–dextran gels containing no enzyme. Each data point represents an average of triplicate measurements, with standard error <15%.

Finally, to further characterize the combined roles of confinement and crowding, we used the 8-anilino-1-naphthalenesulfonic acid (ANS)-binding fluorescence assay to determine the structural stability of enzymes encapsulated in agarose incorporating or not incorporating dextran. ANS is a fluorescent dye that binds to exposed hydrophobic regions of partially folded or fully unfolded proteins, an event that is accompanied by an increase in fluorescence intensity and a blue shift of the emission spectrum [38,39,40]. These experiments clearly revealed a high degree of correlation between the kinetic studies and structural measurements of enzyme stability. Specifically, we observed a significant increase in ANS fluorescence for solution-phase HRP relative to that of HRP encapsulated in agarose (Figure 5). More importantly, we noted higher enhancements in enzyme structural stability (as affirmed by the reduced ANS fluorescence intensity values) for agarose–dextran hybrid gels relative to pure agarose gels (Figure 6). Altogether, our results clearly emphasized the combined roles of confinement and crowding on the functional and structural stability of enzymes.

## 3. Discussion

Research, both theoretical and experimental, over the past couple of decades has underscored the importance of replicating in vivo conditions to study protein structure and function [1,2,3,4,5,6]. Native cellular environments, such as the cytoplasm, are packed with biomacromolecules that subject proteins to highly confined and crowded environments, and these conditions have been shown to impact the equilibria of biochemical processes such as protein folding, as well as protein function and stability [2,3]. Given that traditional experimental protocols for the study of protein fates involved dilute buffer systems and did not replicate the in vivo conditions, new models have been developed to mimic natural confinement and crowding by using macromolecules, mesoporous silica matrices, reverse micelles, or hydrogels [7,8,9,10,11,12,13,14,15,16,17,25]. These studies demonstrated significant effects on several protein fates, including activity and functional stability, their interactions with self and other proteins, and unfolding and aggregation. While there is enough evidence to confirm that confinement and crowding can significantly impact protein properties, it is not yet clear if they function similarly or differently to replicate in vivo cell environments [14,19,41]. Furthermore, only a few studies have explored the combined effects of confinement and crowding, an additional complexity that may arise due to the heterogeneous mixture of differently sized biomacromolecules, typical of native cellular environments [17,24,25,26]. 

The main goal of this study was to explore if we could leverage the hydrogel-based platform previously developed in our lab to further understand the combined effects of confinement and crowding on protein stability and activity [15]. Specifically, we explored the effects of dextran on the stability of the model enzymes, HRP and β-gal, confined within the pores of agarose. Please note that the incorporation of dextran into agarose may influence the average pore size of the gels, and convolute the measurements and the study of the different combinations of confinement and crowding on protein activity and stability. Nevertheless, we believe that the use of agarose–dextran hybrid gels better reproduces the complexity of in vivo environments and is a relevant representation of the combined effects of confinement and crowding on the functional and structural fates of proteins. Our results indicated that the addition of dextran further increased the stability of agarose-confined enzymes, consistent with previous research that demonstrated an increase in the stability of proteins confined in silica pores as a function of crowding [17]. Although Shin et al. reported increases in protein stability using protein crowders (and not polymer crowders), the consistency in findings are not altogether surprising because research has already demonstrated that polymer and protein crowders act similarly on protein stability [42]. It is also important to note that higher relative enhancements in both functional and structural stability were observed for proteins encapsulated in lower concentrations of agarose. The latter result indicates possible saturation in the combined effects of confinement and crowding on protein fates. Our data is consistent with previous research studies that demonstrated greater stabilizing effects for mixed macromolecular crowding, as well as a combination of confinement and crowding [17,22,23]. Furthermore, the observed saturation in protein stabilization, as well as non-additive effects on protein stability, points to a possible synergistic effect of confinement and crowding. We hope this study will provide a foundation to create more relevant models for the in vitro investigation of protein behavior and fates in vivo. Such models may enable exploration of the mechanisms of multidomain protein folding and protein association and interactions, or to develop systems for drug delivery or implantable biosensors [26,43,44,45].

## 4. Materials and Methods 

### 4.1. Materials

The enzymes, horseradish peroxidase (HRP) and β-galactosidase (β-gal), as well as the reagents for quantifying their activity and structure, including the enzyme substrates 2,2′-azinobis(3-ethylbenzthiazoline-6-sulphonate) (ABTS), hydrogen peroxide (H_2_O_2_) (for HRP), and *o*-nitrophenyl β-d-galactoside (ONPG) (for β-gal), and 8-anilino-1-naphthalenesulfonic acid (ANS) were obtained from Sigma-Aldrich (St. Louis, MO, USA). Materials for the confinement and crowding experiments, agarose (molecular biology grade) was obtained from Bio-Rad Laboratories (Hercules, CA, USA) and different molecular weight dextrans (~150 and ~500 kDa)) were purchased from Sigma-Aldrich and used without further purification. All other reagents, including the buffer components, were purchased from Sigma-Aldrich and used as received.

### 4.2. Encapsulation of Enzymes in Agarose Gels

Enzymes were encapsulated in agarose, as described previously [15]. Briefly, stock agarose solutions were first prepared by mixing agarose powder in Tris-HCl buffer mixture at 90 °C for 30 min, and then cooled down to and maintained at 45 °C. Freshly prepared enzyme stock solutions in Tris-HCl buffer were mixed with the agarose solutions at 45 °C, and aliquoted into wells of a 96-well plate (50 μL of the enzyme–gel mixture per well) and cooled to room temperature. Agarose–dextran hybrid gel-encapsulated enzyme formulations were prepared by combining appropriate volumes of agarose, low- or high-molecular-weight dextran, and enzyme stock solutions at 45 °C, before aliquoting into the well plates. Except for the experiments performed to study the role of crowder size on protein stability, high-molecular-weight dextran (~500 kDa) was used to prepare the agarose–dextran hybrid gels.

### 4.3. Measurement of Enzyme Activity

The initial reaction rates of solution-phase and gel-encapsulated enzymes were determined using a Tecan Infinite 200 PRO spectrophotometer (Durham, NC, USA). HRP oxidizes ABTS at the expense of H_2_O_2_ to form a soluble end product that can be spectrophotometrically monitored at 405 nm; HRP concentration for the activity measurements was 90 nM, and the ABTS and H_2_O_2_ concentrations were 10 µM and 40 µM, respectively. β-gal activities were measured by monitoring ONPG hydrolysis at 420 nm; β-gal and ONPG concentrations for these measurements were 10 nM and 80 µM, respectively. Initial reaction rates of ABTS oxidation and ONPG hydrolysis by agarose and agarose–dextran hybrid gels containing no enzyme were measured as controls.

### 4.4. Measurement of Enzyme Functional Stability

For the thermal deactivation (or half-life) experiments, solution-phase and gel-encapsulated enzyme formulations were exposed to elevated temperatures (HRP at 60 °C and β-gal at 50 °C) for different periods of time, followed by cooling to room temperature, before measuring the residual activity. The enzyme activity at zero time (*t* = 0 min) was taken as 100%. For the optimum temperature measurements, activities of the solution-phase and gel-encapsulated HRP were measured at various temperatures (25–70 °C, by 5 °C increments) under aforementioned HRP activity assay conditions. The HRP formulations were first equilibrated at the various temperatures for 5 min prior to the activity measurements.

### 4.5. Measurement of Enzyme Structural Stability

The fluorescence measurements for the ANS assay (used to assess the structural stability of the solution-phase and gel-encapsulated enzymes) were also carried out using the Tecan Infinite 200 PRO spectrophotometer. The HRP formulations were first equilibrated at 60 °C for 50 min prior to the ANS fluorescence measurements. ANS fluorescence emission spectra (between 440 and 540 nm) of the solution-phase and gel-encapsulated HRP were then collected after excitation at 360 nm at room and elevated (60 °C) temperatures. Fluorescence spectra of the agarose or agarose–dextran hybrid gels containing no enzyme were recorded similarly and subtracted from the spectra of enzyme. Please note that agarose and agarose–dextran hybrid gels had minimal interference with the fluorescence measurements.

## Figures and Tables

**Table 1:** Percent increase in half-lives of enzymes encapsulated in agarose–dextran ^a^ hybrid gels (prepared using ~500 kDa dextran) relative to solution-phase enzymes.

	HRP		β-gal	
	100 mg/mL Dextran	200 mg/mL Dextran	100 mg/mL Dextran	200 mg/mL Dextran
Solution phase	29.5 ± 4.9	58.3 ± 5.3	40.1 ± 7.6	76.6 ± 5.6
0.5% Agarose	45.8 ± 6.5	75.2 ± 8.1	73.8 ± 6.3	120.2 ± 6.3
2% Agarose	52.7 ± 5.8	81.9 ± 7.5	93.2 ± 8.0	137.4.1 ± 9.7

^a^ 500 kDa dextran was used for the experimental results reported in Table 1.

**Table 2:** Percent increase in half-lives of enzymes encapsulated in agarose–dextran ^a^ hybrid gels (prepared using low-molecular-weight dextran) relative to solution-phase enzymes.

	HRP		β-gal	
	100 mg/mL Dextran	200 mg/mL Dextran	100 mg/mL Dextran	200 mg/mL Dextran
Solution phase	35.2 ± 4.2	56.1 ± 6.4	49.7 ± 6.1	91.9± 10.7
0.5% Agarose	47.7 ± 6.5	90.2 ± 5.2	81.9 ± 7.5	142.5 ± 8.7
2% Agarose	60.2 ± 5.1	100.2 ± 10.6	96.1 ± 6.8	150.9 ± 9.2

^a^ 150 kDa dextran was used for the experimental results reported in Table 2.

## Supplementary Materials

The following are available online at https://www.mdpi.com/1422-0067/21/22/8516/s1, Figure S1: Thermal stability of HRP and β-gal in dextran solutions.
