# Supplementary material for: Combined Effects of Confinement and Macromolecular Crowding on Protein Stability
Source: Int J Mol Sci. 2020 Nov 12;21(22):8516. doi: 10.3390/ijms21228516 (PMC7697604; doi:10.3390/ijms21228516)
Supplement: Supplementary file 1 [file ijms-21-08516-s001.pdf]

International Journal of
Molecular Sciences

MDPI

Supplementary Information

Combined Effects of Confinement and Macromolecular Crowding on Protein Stability

Murial Ross, Jeffrey Kunkel, Steven Long, and Prashanth Asuri*

Department of Bioengineering, Santa Clara University, Santa Clara, CA 95053, USA; slong1@scu.edu (S.L.); mross@scu.edu (M.R.)

* Correspondence: asurip@scu.edu; Tel.: +1-408-551-3005

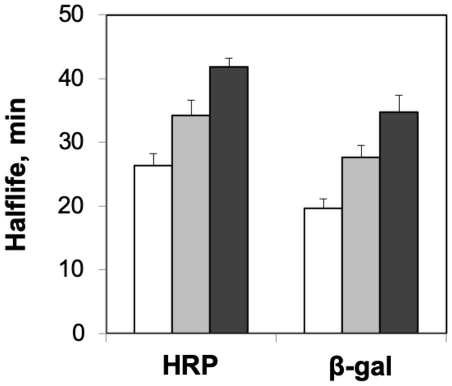

Figure S1. Thermal stability of HRP and β-gal in dextran solutions. Estimated half-lives of solution phase enzymes (white bars) and enzymes in 100 mg/mL (light gray bars) and 200 mg/mL (dark gray bars) dextran at elevated temperatures. Error bars indicate the standard deviation of triplicate measurements.

Methods

Measurement of Enzyme Functional Stability

For the thermal deactivation experiments, solution phase enzymes and enzymes in high molecular weight dextran (~500 kDa) were exposed to elevated temperatures (HRP at 60 °C and β-gal at 50 °C) for different periods of time, followed by cooling to room temperature, before measuring the residual activity. The enzyme activity at zero time (t = 0 min) was taken as 100%.
